# Longitudinal Cell Tracking and Simultaneous Monitoring of Tissue Regeneration after Cell Treatment of Natural Tendon Disease by Low-Field Magnetic Resonance Imaging

**DOI:** 10.1155/2016/1207190

**Published:** 2016-01-10

**Authors:** Dagmar Berner, Walter Brehm, Kerstin Gerlach, Claudia Gittel, Julia Offhaus, Felicitas Paebst, Doreen Scharner, Janina Burk

**Affiliations:** ^1^Large Animal Clinic for Surgery, Faculty of Veterinary Medicine, University of Leipzig, An den Tierkliniken 21, 04103 Leipzig, Germany; ^2^Translational Centre for Regenerative Medicine, University of Leipzig, Philipp-Rosenthal-Straße 55, 04103 Leipzig, Germany; ^3^Institute of Veterinary Physiology, University of Leipzig, An den Tierkliniken 7, 04103 Leipzig, Germany

## Abstract

Treatment of tendon disease with multipotent mesenchymal stromal cells (MSC) is a promising option to improve tissue regeneration. To elucidate the mechanisms by which MSC support regeneration, longitudinal tracking of MSC labelled with superparamagnetic iron oxide (SPIO) by magnetic resonance imaging (MRI) could provide important insight. Nine equine patients suffering from tendon disease were treated with SPIO-labelled or nonlabelled allogeneic umbilical cord-derived MSC by local injection. Labelling of MSC was confirmed by microscopy and MRI. All animals were subjected to clinical, ultrasonographical, and low-field MRI examinations before and directly after MSC application as well as 2, 4, and 8 weeks after MSC application. Hypointense artefacts with characteristically low signal intensity were identified at the site of injection of SPIO-MSC in T1- and T2^*∗*^-weighted gradient echo MRI sequences. They were visible in all 7 cases treated with SPIO-MSC directly after injection, but not in the control cases treated with nonlabelled MSC. Furthermore, hypointense artefacts remained traceable within the damaged tendon tissue during the whole follow-up period in 5 out of 7 cases. Tendon healing could be monitored at the same time. Clinical and ultrasonographical findings as well as T2-weighted MRI series indicated a gradual improvement of tendon function and structure.

## 1. Introduction

Tendon disease is a common musculoskeletal condition in human and equine athletes. Due to their similar properties, the equine superficial digital flexor tendon (SDFT) is considered as an excellent model for the human Achilles tendon [1]. Both tendon structures display an energy storage function, and peak tendon loads during locomotion amount to several thousand newtons [2, 3]. Moreover, in the equine SDFT, overload- and strain-induced disease occurs naturally and displays similar ultrasonographic, magnetic resonance imaging (MRI) and histopathological changes as described for the human Achilles tendon [1, 3]. In both human and equine patients, therapy of tendon disease remains challenging, leading to research on regenerative therapies to improve the clinical outcome. In horses, treatment of tendon disease with mesenchymal stromal cells (MSC) has been performed in the last decades with promising results [4, 5], potentially representing a therapeutic option for human medicine as well.

MSC are adult progenitor cells which are characterized by their plastic-adherence and multipotent differentiation potential in vitro as well as by a set of positive and exclusion antigen markers [6]. MSC can be recovered from different tissues and fluids in the body, including birth-associated tissues, and are known to display diverse properties depending on the tissue source [7, 8]. This indicates the importance of choosing the appropriate source of MSC for therapeutic applications. In this study, we aimed to use allogeneic MSC in order to be able to provide vital and consistent cell populations for all patients within a short period of time after their presentation. For this purpose, we chose to use umbilical cord-derived MSC, as their allogeneic application in the horse had best been investigated at the time the current study was initiated [9] and could be considered as safe. Moreover, the umbilical cord is known to provide high numbers of vital MSC with superior proliferation potential compared to other sources such as umbilical cord blood or bone marrow [10, 11]. In addition to that, own studies had demonstrated that umbilical cord-derived MSC, while displaying multilineage differentiation potential, deposited less mineralizations during osteogenic differentiation compared to MSC from other sources [8], which potentially minimizes the risk of calcification after MSC treatment of tendon disease.

Experimental and clinical case studies in the equine model suggest that an improved rearrangement of tendon collagen fibers after local application of MSC leads to reduced reinjury rates compared to conventional treatment [4, 12–17]. However, the exact mechanisms underlying the therapeutic benefit of MSC application are still unclear. It still remains to be elucidated whether the enhancement in tissue regeneration is due to cell replacement by the implanted MSC or whether it is driven by the host cell response to the implanted MSC. Additionally, the exact fate of implanted MSC and their degree of survival as well as their potential integration into newly formed tissue are still under investigation.

Therefore, gaining more insight into the fate of transplanted MSC is a crucial step for understanding the complex repair mechanisms. In the equine tendon disease model, cell tracking was first attempted using green fluorescent protein-labelled MSC and histological assessment of the tendons after sacrificing the horses at day 10 or day 34. In this study, it was shown that labelled cells could mainly be found at the injection site [18]. Furthermore, scintigraphic tracking of MSC labelled with technetium was performed after direct intralesional, intravenous, or intra-arterial injection, demonstrating that intralesional injection resulted in the highest MSC uptake in the tendon lesion during the assessment period of 24 or 48 h [19–21]. While these studies provided important information, both cell tracking techniques have their limitations and cannot be used for longitudinal in vivo cell tracking: histology of representative tendon specimen can only be performed at one time point and after euthanasia of the animals, while scintigraphic tracking using technetium is only feasible over a relatively short time frame, requires radioactive cell labelling, and has a lower spatial resolution than MRI.

Labelling of MSC with superparamagnetic iron oxide (SPIO) particles, which are incorporated by the cells via endocytosis, is an alternative technique which enables tracking the labelled cells noninvasively by MRI [22–26]. The paramagnetic substances induce inhomogeneities in the magnetic field and cause susceptibility artefacts. In vitro studies have shown that those hypointense artefacts are best seen in T2^*∗*^-weighted (T2^*∗*^w) gradient echo (GRE) images. Moreover, the artefacts could be seen in vitro for up to 21 days after labelling [22, 27, 28], indicating that this technique could be useful for longitudinal in vivo tracking. Besides the possibility of long-term tracking of SPIO-labelled cells, the use of MRI offers the major advantage that the surrounding tissue structures can be visualized at the same time and with good accuracy. Especially in case of tendon and ligament disease, MRI is a gold standard technique for diagnosis and monitoring of the healing process, which makes it an attractive tool for cell tracking after cell treatment of tendon disease. In sheep, mice, rats, and rabbits, first in vivo studies have investigated the distribution of SPIO-labelled cells in musculoskeletal tissues, which could be visualized for several weeks by MRI [24, 26, 29, 30].

Using the equine model, there is the additional advantage that these animals can be subjected to MRI examinations of their distal limbs without general anesthesia, due to the availability of a specialized low-field MRI system established for equine patients. This allows for performing follow-up examinations at close time intervals and with minimal risk and stress exposure for the animals. However, in the equine large animal model, only an ex vivo study evaluating SPIO-labelled MSC by MRI in the distal limb has been performed so far [31].

The aim of this study was to perform the first long-term tracking of MSC applied for treatment of natural tendon disease. It was further aimed to monitor tendon healing and simultaneously localize the injected MSC at different time points by use of the equine dedicated low-field MRI system. In addition to that, the compatibility of allogeneic umbilical cord-derived MSC for treatment of tendon disease was to be evaluated.

## 2. Materials and Methods

### 2.1. Cell Recovery

Umbilical cord tissue was obtained from 2 warmblood foals immediately after birth. Nuclear cells were isolated by collagenase digestion as described previously [8], and the plastic-adherent fraction was expanded in low glucose (1 g/L) Dulbecco's modified Eagle medium (DMEM; Invitrogen, Darmstadt, Germany) supplemented with 20% fetal bovine serum (FBS; Sigma Aldrich, Munich, Germany), 100 I.U./mL penicillin, 0.1 mg/mL streptomycin (1% penicillin-streptomycin, PAA Laboratories GmbH, Coelbe, Germany), 0.05 mg/mL gentamycin (Invitrogen), and 0.5 *μ*g/mL amphotericin (Invitrogen) under standard culture conditions (37°C, 5% CO_2_). At 80% confluency, cells were passaged and portions of the obtained cells were cryopreserved, while the remaining cells were used for cell characterization at passage 3. Cryopreservation was performed in a medium containing 40% DMEM (Invitrogen), 40% FBS (Sigma Aldrich), and 20% dimethyl sulfoxide (Sigma Aldrich), and in a container with a cooling rate of −1°C/min. MSC were then stored in liquid nitrogen until further use. Cell characterization included trilineage differentiation assays as well as analysis of tendon and MSC marker expression as described previously [8, 32, 33].

### 2.2. Cell Preparation and Labelling

Prior to clinical use, the cryopreserved MSC were thawed, seeded onto culture plates, and incubated under standard conditions as described above. At 80% confluency, MSC were labelled with SPIO-rhodamine particles (Molday ION Rhodamine B, BioPAL Inc., Worcester, MA, USA) at an iron concentration of 25 *μ*g/mL [22] for 16 h for application in the cell tracking group. After labelling, cells were thoroughly washed and trypsinized. MSC were then suspended in the patients' freshly prepared serum (10 × 10^6^ MSC per mL) and were applied within 2 h after preparation.

For confirmation of successful labelling, part of the labelled cells was seeded in 12-well plates and stained with Prussian blue to visualize the intracellular SPIO particles by light microscopy and with DAPI counterstaining of nuclei for rhodamine visualization by fluorescence microscopy. Additionally, labelled MSC were further cultured and examined by MRI 1, 2, and 4 weeks after labelling. For this purpose, gel phantoms containing the labelled MSC were prepared at each time point. First, a 2% agarose (Carl Roth, Karlsruhe, Germany) gel was prepared and 0.2 mL tubes were placed beneath the surface to create wells in the gel while it was hardening. MSC were suspended in a 1% agarose gel at concentrations of 10^6^, 10^5^, or 10^4^ MSC per 50 *μ*L and pipetted into the wells. Finally, the wells containing the MSC were again covered with 2% agarose. T1-weighted (T1w) and T2^*∗*^w GRE images of the gel phantoms were obtained by low-field MRI.

### 2.3. Animals

Equine patients presenting with naturally occurring SDFT disease were recruited for this study. Inclusion in the study was based on the ultrasonographic diagnosis of a hypoechoic SDFT lesion at the metacarpal region in at least one forelimb. Nine animals (5 warmbloods, 2 ponies, 1 Haflinger, and 1 thoroughbred; 8 geldings, 1 mare; and 2 jumping horses, 3 dressage horses, and 4 pleasure horses; mean age: 14 years, age range: 4–23 years) met the inclusion criteria and 11 SDFT lesions could be subjected to cell treatment and were included in the study. Two-thirds of the SDFT were randomly assigned to the cell tracking group and treatment with labelled MSC, while the remaining SDFT were treated with nonlabelled MSC to serve as controls for the evaluation of cell tracking results (Table 1). The mean period of time between onset of clinical signs and cell injection was 28 days (range: 18–20 days). All procedures were performed at the Large Animal Clinic for Surgery, University of Leipzig, with informed consent of the horses' owners and in accordance with the local Animal Care and Use Committee requirements (Landesdirektion Leipzig, TV 34/13).

### 2.4. Cell Injection and Patient Management

For MSC application, the skin was clipped and prepared aseptically. In the standing, sedated horse, 10 × 10^6^ MSC suspended in 1 mL of serum were injected into each tendon lesion under ultrasonographic guidance with a 23 G needle centred into the lesion. Sedation was achieved as described below. Thereafter, the distal limb was bandaged.

During the acute phase of tendon disease and for one further week following the MSC application, the horses were restricted to stall rest. During that time, they were also administered nonsteroidal anti-inflammatory drugs (NSAID) when showing signs of pain (flunixin-meglumine 1.1 mg/kg bwt p.o., CP-Pharma Handelsgesellschaft mbH, Burgdorf, Germany). After the period of stall rest, patients were subjected to a gradually increasing, controlled exercise program suggested previously [4].

### 2.5. Follow-Up and Diagnostic Imaging

Follow-up examinations were designed to monitor potential clinical side effects of MSC application and tendon healing as well as the distribution of the applied MSC. The equine patients were subjected to clinical, ultrasonographical, and MRI examinations directly prior to MSC injection as well as 2, 4, and 8 weeks after MSC injection. For cell tracking purposes, MRI was additionally performed directly after cell application.

Clinical examination included the evaluation of swelling, pain to palpation, and signs of pain when walking and trotting (lameness). Lameness was graduated according to the established scale provided by the American Association of Equine Practitioners (AAEP), which ranges from 0 (no lameness) to 5 (profound lameness with minimal weight bearing).

Ultrasonographic examinations of the SDFT were performed in transverse and longitudinal plane using a 10 MHz linear transducer (LOGIQ 5 Expert, GE Healthcare, Munich, Germany) with a standoff probe. The SDFT was divided into 7 zones (1a-3c) [34] and at least one image was recorded at every level.

**Figure 1:**
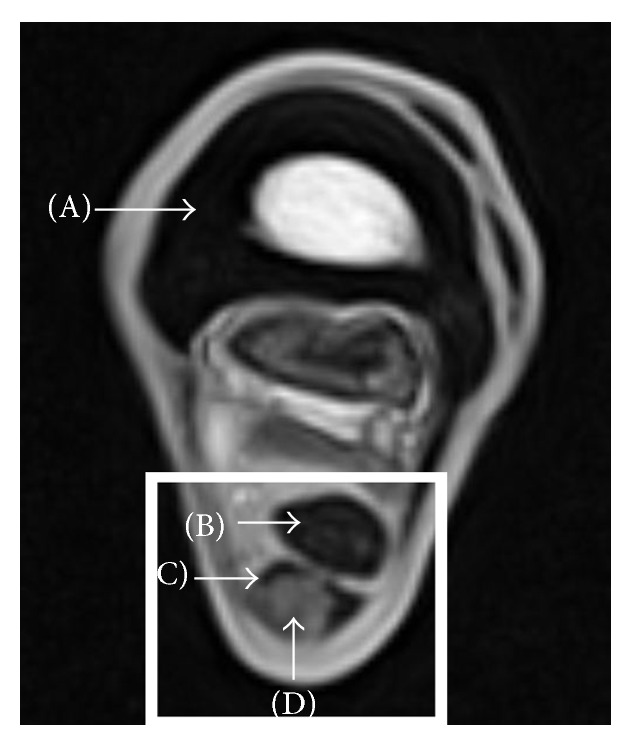
Exemplary transverse T1-weighted gradient echo magnetic resonance image of the equine distal limb in the metacarpal region, with arrows indicating the metacarpal bone (A), the hypointense deep digital flexor tendon (DDFT, B), and the superficial digital flexor tendon (SDFT, C) displaying a hyperintense intratendinous lesion (D) surrounded by hypointense healthy tendon tissue. The rectangle marks the region relevant to this study, which is displayed in all other figures; lateral is displayed on the left.

MRI of the injured SDFT region was performed using a 0.27 Tesla dedicated equine low-field MRI system (Hallmarq EQ2, Hallmarq Veterinary Imaging, Guildford, Surrey, UK). Horses were sedated with romifidine hydrochloride (0.04 mg/kg bwt i.v., Sedivet, Boehringer Ingelheim Vetmedica GmbH, Ingelheim am Rhein, Germany) and butorphanol tartrate (0.02 mg/kg bwt i.v., Alvegesic, CP-Pharma Handelsgesellschaft mbH, Burgdorf, Germany). Sedation was maintained as required either with detomidine hydrochloride (20 mg in 500 mL NaCl i.v., Cepesedan, CP-Pharma Handelsgesellschaft mbH) or with romifidine hydrochloride (20 mg in 500 mL NaCl i.v.) in combination with butorphanol tartrate (10 mg in 500 mL NaCl i.v., Alvegesic, CP-Pharma Handelsgesellschaft mbH). T1w and T2^*∗*^w GRE, T2-weighted (T2w) fast spin echo sequences (FSE), and short tau inversion recovery (STIR) FSE sequences were acquired in transverse plane (Table 2). An exemplary MRI image depicting the relevant anatomical structures is shown in Figure 1.

### 2.6. Image Analysis

All 55 ultrasonographic studies and MRI series obtained from the 11 SDFT at the 5 separate examinations were randomized and evaluated in consensus by two observers blinded to examination date as well as group status (Synedra ViewPersonal, version 3.4.0.2, Synedra Information Technologies GmbH, Innsbruck, Austria).

Tendon healing was evaluated in MRI series and ultrasonographic images, with the latter representing the current standard technique for diagnosis and monitoring of SDFT disease.

Ultrasonographic images were first evaluated semiquantitatively, using a score system which included lesion echogenicity (grades 1 to 3; 1 = mildly hypoechoic, 2 = moderately hypoechoic, and 3 = anechoic), presence of peritendinous edema (grades 0 to 3; 0 = none, 1 = minimal, 2 = moderate, and 3 = severe), and fiber pattern (grades 1 to 3; 1 = mildly irregular, 2 = moderately irregular, and 3 = fiber disruption) as described previously [35]. The final score was calculated by summarizing all points assigned to the individual parameters. For quantitative measurements, ultrasonographic images at the level of maximum injury were selected and used to determine the percentage of the lesions within the SDFT (LE % = lesion cross-sectional area/tendon cross-sectional area × 100).

Analysis of tendon healing in MRI series was also performed using images obtained at the level of maximum injury, which was determined based on T1w series. In the other sequences, corresponding images were used. Quantitative measurements in MRI images included the LE % of each tendon lesion as described above. Furthermore, the signal intensities of the lesion (SI LE) and the unaltered deep digital flexor tendon (SI DDFT) were obtained by measuring the signal intensity of a circular region of interest (ROI) in the respective structure, which was then normalized to the background noise obtained from a circular ROI outside the limb within the same image.

Identification of labelled cells was attempted by evaluation of corresponding MRI sequences of each separate examination. Presence or absence of hypointense artefacts potentially corresponding to injected SPIO-labelled cells was subjectively assessed in all randomized MRI series. Hypointense areas within the tendon lesion or surrounding tissues were considered as SPIO-induced artefacts when being present on T2^*∗*^w GRE images but not on the corresponding T2w and STIR FSE images which showed high lesion signal intensity irrespective of the injection. If such artefacts were present, further quantitative analysis was performed in T1w and T2^*∗*^w GRE images. The signal intensity of the potentially SPIO-related hypointense artefacts (SI SPIO) and the signal intensity of the unaltered DDFT (SI DDFT) were measured using a circular ROI and then normalized to the background noise obtained from a circular ROI outside the limb within the same image. Additionally, the areas covered by the hypointense artefacts were measured.

Furthermore, subsequent to the blinded evaluation, the MRI images were reevaluated to further characterize the hypointense artefacts found after injection of SPIO-MSC. For this purpose, the unblinded MRI images of the different examinations were assessed chronologically. During this second assessment, besides the presence of artefacts, their localization within the tendon and surrounding tissues was evaluated.

### 2.7. Statistical Analysis

Measurements during image analysis were repeated three times and the mean values were used for statistical analysis. Using SPSS 22 statistics software (IBM, Ehningen, Germany), Friedman tests were performed to analyze differences between different examination times or between different imaging modalities and MRI sequences. In case of significance, Wilcoxon rank-sum tests were applied subsequently. *P* values < 0.05 were considered as significant.

## 3. Results

### 3.1. Cell Recovery and Characterization

MSC could be recovered without complications from both samples. The cells were plastic-adherent, highly vital, and capable of adipogenic, osteogenic, and chondrogenic differentiation. The cells were also shown to express the tendon markers collagen 1A2, collagen 3A1, decorin, tenascin-C, and scleraxis on mRNA level as well the MSC-related antigens CD 29, CD 44, and CD 105 on protein level.

### 3.2. Cell Labelling

**Figure 2:**
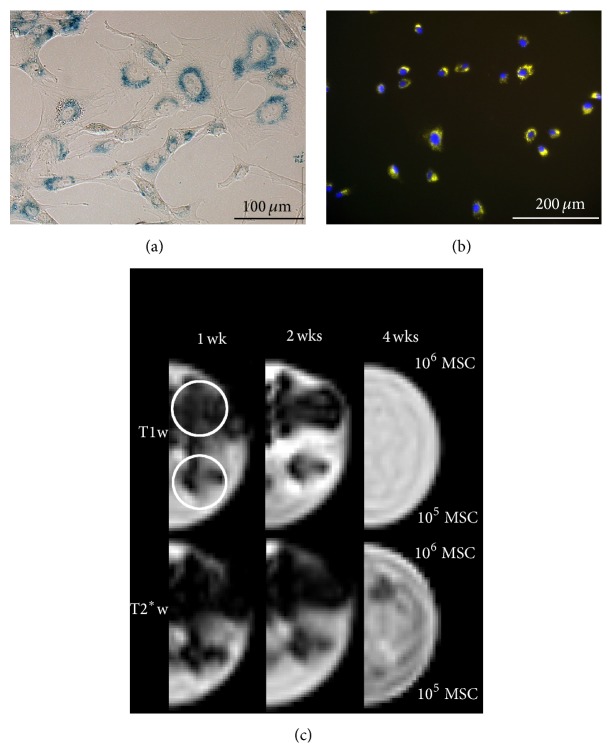
Microscopic and magnetic resonance images of labelled cells. Prussian blue staining of intracellular iron oxide particles (a); orange fluorescence of the intracellular rhodamine, nuclei in blue (b); T1-weighted (T1w) and T2^*∗*^-weighted (T2^*∗*^w) gradient echo (GRE) images (c) of the gel phantoms 1 week (1 wk), 2 weeks (2 wks), and 4 weeks (4 wks) after cell labelling. The upper wells in each gel contain 10^6^ labelled cells (MSC), the lower wells 10^5^ labelled cells (MSC). The shape of the wells is indicated exemplarily by circles in the first upper image in (c). The hypointense artefacts caused by the labelled cells are visible in both sequences until 2 weeks after labelling. Here, the higher cell concentration led to artefacts exceeding the shape of the well in which the MSC were localized. Four weeks after labelling, no artefacts were seen in the T1w GRE sequence and only 10^6^ MSC induced weak hypointense artefacts in the T2^*∗*^w GRE sequence.

Intracellular uptake of the SPIO-rhodamine labelling substance could be confirmed microscopically (Figure 2). MRI of the gel phantoms showed that the labelled cells were visible as hypointense artefacts in low-field MRI. In T2^*∗*^w GRE images, 10^6^ MSC were visible until week 4 and 10^5^ MSC were visible until week 2, although the artefact area decreased over time. In T1w GRE images, 10^6^ and 10^5^ MSC were visible until week 2 and the artefact was overall less pronounced than in the T2^*∗*^w GRE images. Detection of 10^4^ MSC was not possible reliably by low-field MRI. Furthermore, it was noticeable that MRI displayed an artefact area exceeding the true localization of MSC in the round well when they were highly concentrated (Figure 2).

### 3.3. Clinical Findings and Compatibility of Umbilical Cord-Derived MSC

Mild to moderate lameness was observed at the day of presentation in all horses when trotting (grades 1–3/5 according to AAEP scale). The lameness score decreased in all horses over the study period of 8 weeks, indicating an improvement of tendon function. At week 8, lameness was completely absent in 4 horses; in the remaining horses, only mild lameness was visible when trotting (grade 1/5 according to AAEP scale). The injection of MSC was tolerated without any subsequent side effects in 6 out of 9 animals. The remaining horses, which were those suffering from more severe tendon disease, showed a moderate but transient local reaction with pain to palpation, local swelling, and an increase in lameness. However, these side effects were manageable with NSAID and resolved within one week.

### 3.4. Imaging of Tendon Healing

**Figure 3:**
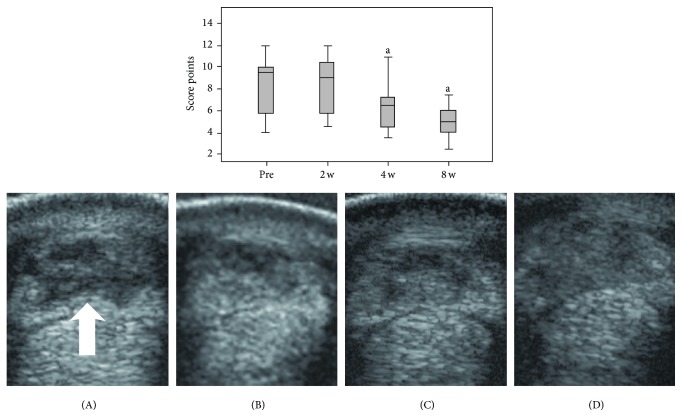
(Up): boxplot of score points obtained in ultrasonographic assessment in all cases at the day of treatment (pre) and 2 weeks (2 w), 4 weeks (4 w), and 8 weeks (8 w) after treatment, with “a” indicating lower score points (improvement) compared to 2 w (*P* < 0.05). (Down): exemplary cross-sectional ultrasonographic images of a severe tendon lesion with an extensive hypoechoic defect (arrow) of the superficial digital flexor tendon at the day of treatment (A), 2 weeks after treatment (B), and 4 (C) and 8 (D) weeks after treatment when showing gradual filling of the defect and decrease of tendon enlargement.

The score points obtained after semiquantitative assessment of ultrasonographic images decreased over time, demonstrating progress in tendon regeneration. While score points obtained before injection and 2 weeks after injection were still similar, the decrease was evident at 4 and 8 weeks (*P* < 0.05 compared to week 2) (Figure 3).

**Figure 4:**
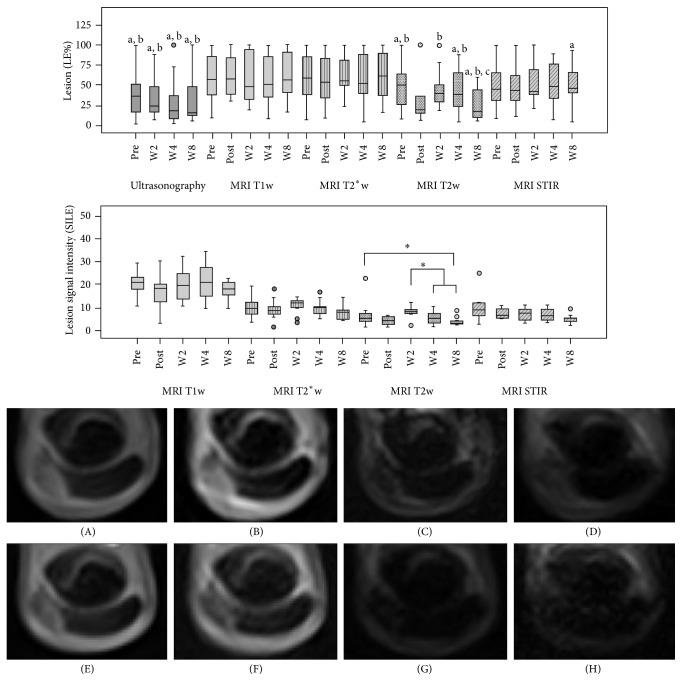
Boxplots displaying the lesion percentage and lesion signal intensity quantified by ultrasonography and/or magnetic resonance imaging (MRI) in all cases and exemplary transversal MRI images from the different examinations before (pre, A–D) and directly (post) as well as 2 (2 w), 4 (4 w), and 8 (8 w, E–H) weeks after cell application. Displayed MRI images are T1-weighted gradient echo (T1w, A + E), T2^*∗*^-weighted gradient echo (T2^*∗*^w, B + F), T2-weighted fast spin echo (T2w, C + G), and short tau inversion recovery fast spin echo sequences (STIR, D + H). In the boxplots, “a,” “b,” and “c” indicate lower values compared to T1w, T2^*∗*^w, or STIR images, respectively (*P* < 0.05); stars indicate differences between T2w images from different examinations. The MRI images demonstrate the decrease in signal intensity and lesion percentage in the T2w and STIR sequences; lateral is displayed on the left.

LE % measured on ultrasonographic images gradually decreased over time as well, although no significant differences could be observed. Similarly, no significant differences were evident regarding LE % obtained from MRI series over time. However, while the values from T1w GRE, T2^*∗*^w GRE, and STIR FSE sequences remained similar, T2w FSE images displayed variations. Here, an overall decreasing trend was observed in LE % in T2w FSE images until week 8, corresponding to the ultrasonographic findings. Furthermore, a transient decrease in LE % was observed directly after cell injection, although this was presumably due to air infiltration during the injection procedure, which may have hampered correct identification of the lesion. More significant differences regarding LE % were observed when comparing the different MRI sequences and ultrasonographic images obtained at the same examinations. LE % measured in ultrasonographic and T2w FSE images was lower compared to LE % obtained from T1w and T2^*∗*^w GRE images. This was not only evident at the later examinations when LE % had decreased in ultrasonographic and T2w FSE images, but also at the early examinations (*P* < 0.05 before injection and at weeks 2, 4, and 8). At week 8, T2w FSE LE % was additionally lower than STIR FSE LE % (*P* < 0.05) (Figure 4).

Corresponding results were obtained when measuring the MRI signal intensities. Before injection, all tendon lesions showed high signal in all MRI sequences and were hypoechoic in ultrasound images. Indicating progress in tendon healing, MRI SI LE was lower at week 8 compared to the examinations before cell application and at week 2, which was again most evident in T2w images (*P* < 0.05). Despite this overall decreasing trend, a transient increase of SI LE was observed at week 2. This result corresponded to the clinical findings and was most pronounced in the animals that had presented with a local reaction to cell application. Furthermore, a slight transient decrease in SI LE was observed directly after cell injection in all sequences, most likely representing an artefact again due to air infiltration during the injection process. In contrast to the differences observed for SI LE, SI DDFT, which was used as a control, remained constant over time.

### 3.5. Cell Tracking

**Figure 5:**
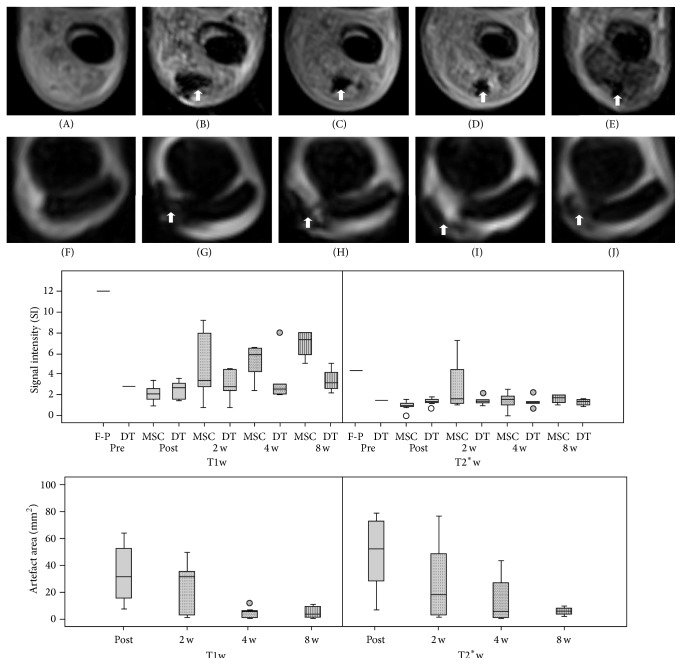
Exemplary transverse T2^*∗*^-weighted gradient echo images of a horse with severe tendinopathy (upper row, A–E) and a horse with moderate tendinopathy (lower row, F–J) that were both treated with labelled cells, and boxplots displaying the signal intensities of regions of interest measured for cell tracking and the areas covered by hypointense artefact in T1-weighted gradient echo (T1w) and T2^*∗*^-weighted gradient echo (T2^*∗*^w) images. All separate examinations before injection (A + F, pre) and directly (B + G, post) as well as 2 (C + H, 2 w), 4 (D + I, 4 w), and 8 (E + F, 8 w) weeks after injection are displayed. Before injection, no hypointense areas were visible within the lesion except for 1 case. Directly after injection, hypointense artefacts were located within the lesion and surrounding tissue (arrows). At week 2, week 4, and week 8, the hypointense areas were decreasing gradually but still visible. Furthermore, in the case displayed in the lower row, the artefacts appeared to be located more within the tendon lesion at week 8 compared to week 2 or week 4. Lateral is displayed on the left. The upper boxplot illustrates that signal intensities within hypointense areas relatable to labelled cells (MSC) were low in all cases and mostly within the same range as signal intensity of the corresponding healthy deep digital flexor tendon (DT). The plot also displays the one case in which a false-positive judgement had been made (F-P), showing that this case could be discriminated by the higher signal intensity within the area that had been mistaken for being induced by labelled cells. The lower boxplot shows the decreasing trend in the area covered by hypointense artefacts.

**Figure 6:**
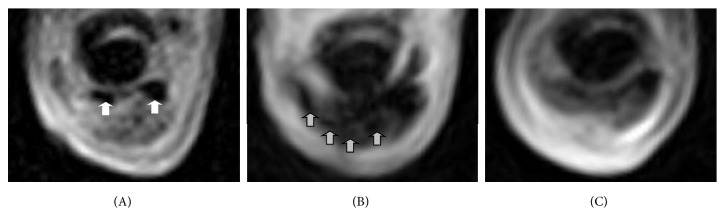
Exemplary transverse T2^*∗*^-weighted gradient echo images of a tendon lesion in which labelled cells could not be discriminated until week 8, before (A) and directly after injection of labelled cells (B) and after 8 weeks (C). The image obtained before injection displays the tendon lesion as well as adjacent hypointense tendon fibres (white arrows). Directly after injection, the hypointense artefacts induced by the labelled cells are clearly visible as additional hypointense areas (grey arrows). After 8 weeks, it was not possible to discriminate these hypointense artefacts from the adjacent hypointense tendon fibers anymore.

By blinded evaluation, in all 7 SDFT lesions that were injected with labelled MSC, corresponding hypointense artefacts could be identified on T1w and T2^*∗*^w GRE images directly after MSC injection. On T2w and STIR FSE images or any images obtained from the control lesions injected with nonlabelled MSC, no such hypointense areas were visible at any examination. The artefacts found after injection of labelled cells showed a decrease of the area covered and an increase of signal intensity in all cases over time but could still be distinguished in 6 of the 7 SDFT at week 2, in 5 of the 7 SDFT at week 4, and in 4 of the 7 SDFT at week 8. In the remaining cases, a clear distinction between hypointense areas caused by the SPIO particles from the hypointense healthy tendon tissue was not possible anymore, leading to potentially false-negative results in blinded subjective evaluation (Figures 5 and 6). No false-positive results were obtained by blinded subjective judgement, except in 1 out of all 55 assessed MRI series. In this one MRI series, which had been acquired before cell application, hypointense healthy tendon tissue within the lesion had been mistaken for a SPIO-induced artefact. This was revealed after completion of image analysis when the image series were assigned back to the corresponding patients and dates. In this case, data obtained by quantitative image analysis were useful to recognize the false-positive subjective judgement. In MRI series of SDFT lesions in which the identified hypointense areas could be related to labelled cells, values of SI SPIO were low and similar to the corresponding SI DDFT. This applied to T2^*∗*^w GRE images from all follow-up examinations as well as to T1w GRE images from the examinations directly and 2 weeks after cell injection. In contrast, the SI value of the hypointense area that had been mistaken for a SPIO-induced artefact was more than 4-fold higher than the corresponding SI DDFT in the T1w GRE image and roughly 3-fold higher in the T2^*∗*^w GRE image. Based on that, especially using the data obtained from T1w GRE images, the SPIO-induced artefacts could be distinguished from the SPIO-unrelated hypointense area (Figure 5).

By unblinded evaluation of the MRI images and comparison to the preceding image series, the hypointense artefacts could be distinguished in fewer cases than those by blinded evaluation (in 6 of 7 SDFT at week 2 and week 4; 5 of 7 SDFT at week 8). During the whole follow-up period, the hypointense artefacts were localized at the level of maximum tendon injury in 5 of the 7 SDFT; in the remaining 2 SDFT, the artefacts were mainly found slightly distal to this level. Furthermore, artefacts were seen in several subsequent transverse images, potentially indicating a distributing of MSC to regions proximal and distal to the injection site. Regarding the cell distribution in transverse plane, the hypointense artefacts were visible within the tendon lesion in all SDFT directly after MSC injection. Distribution within the tendon lesion was variable but included the centre of the lesion in 6 out of 7 cases. Partial potential leakage of cells was observed in 5 cases, with hypointense areas being additionally visible in the surrounding tissue. In the surrounding tissues, artefacts were present until week 8 in 2 cases only. However, artefacts within the lesion remained there until week 8, as far as they were still traceable, except for 1 case in which the artefacts were mostly found within the peritendineum at weeks 2, 4, and 8 (Figure 5). Interestingly, in this case, the localization of the artefact appeared to slightly shift back towards the tendon lesion over time, potentially indicating that the labelled MSC migrated within the injured tissue (Figure 5). However, there was no evidence of extension of the susceptibility artefacts to more distant locations.

## 4. Discussion

In this study, we could demonstrate that noninvasive, longitudinal cell tracking and simultaneous monitoring of tendon healing are feasible by the use of low-field MRI. Hypointense artefacts were present within the tissue after intralesional injection of SPIO-labelled cells into damaged tendons in all cases. They further remained visible at the injection site for the whole follow-up period of 8 weeks in 5 out of 7 cases. While T1w and T2^*∗*^w GRE sequences were useful for cell tracking, T2w FSE MRI series were useful to document the early phase of tendon healing, with the obtained data corresponding to those obtained by the standard ultrasonography technique.

Besides demonstrating the feasibility of cell tracking and simultaneous monitoring of tissue regeneration, this study also showed that the application of SPIO-labelled allogeneic umbilical cord-derived MSC was well tolerated by the animals. Except for moderate and transient local reactions that were observed shortly after the injection in few of the animals, no side effects were observed. Interestingly, such local reactions were only observed in horses with severe tendinopathies, regardless of the treatment group; thus, they were not likely to be associated with SPIO-labelling. This is in accordance with previous studies, in which no unwanted side effects of the application of SPIO-labelled cells in vivo were described [23, 26]. Furthermore, previous studies showed that no major effects on MSC properties due to SPIO-labelling are to be expected [26, 28, 31]. However, a decrease in chondrogenic differentiation capacity and an increase in cell doubling time were evident in SPIO-labelled equine MSC [28, 31]. In these studies, the observed effects could have been induced by the use of high iron concentrations for cell labelling that have previously been shown to negatively influence MSC [22]. To avoid a negative impact of SPIO-labelling on MSC properties, we therefore used a lower iron concentration for the labelling procedure, which had previously been shown to have less influence on MSC but still resulted in a high contrast-to-noise ratio in MRI [22]. With regard to the local reactions, it should further be acknowledged that this may have been due to immune responses to the allogeneic cells. However, transient local reactions have been described after intradermal application of allogeneic as well as autologous umbilical cord-derived MSC but were not accompanied by measurable immune responses or systemic reactions [9]. Furthermore, intratendinous application of autologous MSC was already observed to induce transient local reactions in some cases as well [17]. Based on these previous studies, the current finding is unlikely to be related to the allogeneic treatment. However, while this study suggests a good compatibility of allogeneic umbilical cord-derived MSC for treatment of tendon disease, further studies are necessary to evaluate their efficacy.

The methods applied in this study were feasible for a first long-term tracking of MSC applied for treatment of natural tendon disease according to our aim. The blinded evaluation of randomized MRI images regarding the presence of putatively SPIO-related artefacts demonstrated sensitivity and specificity of the technique, as most of the artefacts were distinguished correctly. Nonetheless, for cell tracking purposes, unblinded chronological image assessment gives more insight into the location of artefacts over time. This is of high importance for future studies, as the technique can be used to monitor MSC localization over time, which on the one hand gives insight into the general behaviour of these cells in vivo and on the other hand can help to improve application techniques. The standard approach used in the current study, injecting the MSC centrally into the tendon lesion under ultrasonographic guidance, already led to relatively good results in terms of distribution of MSC within the injured area, given that the artefacts represent the cells. However, in most cases, it was not achieved to infiltrate the whole lesion area, and additional hypointense areas in the tissue surrounding the tendon suggested partial leakage of the MSC as well. Here, it would be valuable to achieve an optimal cell distribution within the lesion and without leakage, which could be attempted by comparing different volumes or numbers of injection sites, the use of different cannulas, or ultrasonographic versus MRI guided injections.

However, there are some aspects regarding the technique that might be improved in future studies. The fact that, in 2 cases, SPIO-labelled MSC could not be monitored over the whole follow-up period requires further investigation. It could either be due to insufficient presence of SPIO-labelling or due to shortcomings regarding the MRI technique applied. Furthermore, MSC might have distributed within the tendon lesion, resulting in diluted cell concentrations below the detection limit within all examined regions, or they might have migrated to a more distant location that was not examined by MRI. However, the latter is unlikely as no shift in SPIO localization over distances longer than few millimetres was observed in any of the image series obtained.

Giving some insight into the presence of SPIO-labelling, in the gel phantoms, we were able to detect 10^5^ MSC for up to 2 weeks and 10^6^ MSC for up to 4 weeks of in vitro culture by low-field MRI. In another study investigating SPIO-labelling of equine MSC, the cells showed detectable intracellular iron for up to 15 days of in vitro culture [28]. However, MSC rapidly divide in vitro, leading to a continuous loss of intracellular SPIO-particles [31, 36], which complicates a direct comparison of detectability by MRI after in vitro culture and in vivo application. In the current study, after only 1 week of in vitro culture, 10^5^ MSC could be detected by low-field MRI, while lower cell numbers could not be identified reliably. Based on that, it can be assumed that the hypointense artefacts observable in vivo were induced by at least the same number of labelled MSC. Therefore, with 10^5^ MSC representing 1% of the total injected cells per lesion, it can be hypothesized that at least 1% of the injected cells were still present after 8 weeks, in at least 5 out of 7 cases. This is roughly in accordance with another study, in which survival rates of injected MSC in damaged equine SDFT were observed to be less than 1% after 90 days [37]. However, compared to other cell tracking techniques such as scintigraphy, it is a limitation of using MRI for cell tracking that quantifying the exact amount of remaining MSC at the injection site is not possible [19–21].

Assessment of labelled MSC placed in the gel phantom also showed that the area covered by hypointense artefact in MRI images can exceed the actual localization of labelled cells. However, this applied only to the high concentration of 10^6^ MSC per 50 *μ*L. Such high concentrations were unlikely to be found after in vivo application in the current study, as cells were suspended at lower concentrations for the injection on the one hand and further dilution within the tissue can be assumed on the other hand. Therefore, this effect presumably did not have significant impact on the in vivo findings of the current study, but it should be taken into account especially when experimenting with higher cell concentrations.

To confirm the presence of viable, labelled MSC in the current study, histopathological examinations would have been helpful but were not feasible due the use of equine patients which were client-owned horses. Nevertheless, other in vivo studies could confirm the presence of intracellular SPIO-labelling histologically after the in vivo tracking was completed. In a rabbit model of tendon disease, SPIO-labelled MSC could be detected for up to 3 weeks within the tendon by MRI as well as histology [26]. Moreover, follow-up after intra-articular injection of 10 × 10^6^ SPIO-labelled MSC in sheep demonstrated the survival of SPIO-labelled cells for up to 12 weeks by histology [23]. These results are in contrast to another study using a sheep model of tendon disease, in which SPIO-labelled cells could be detected for up to 7 days only, although hypointense signal caused by free SPIO particles was detected for up to 14 days by MRI [25]. However, in this study, only 0.5 × 10^6^ or 1 × 10^6^ MSC were injected into the artificial tendon lesions, which could explain the lack of detectability over a longer period of time.

Regarding the imaging technique applied in the current study, while offering the major advantage that no general anesthesia is required, the use of low-field MRI may have led to the loss of detectability of labelled cells in 2 out of 7 cases. The detection limit for SPIO-labelled MSC is the same in high- and low-field systems [38]. However, to get a reasonable signal-to-noise ratio, a relatively wide slice thickness has to be chosen for the examination of standing horses in low-field systems, decreasing the spatial resolution. In a high-field system, a better spatial resolution with potentially more accurate detection of MSC would have been possible. However, due to the risk and burden of repeated general anesthesia required for multiple high-field MRI examinations in animals, the use of low-field MRI still appears advantageous for continuous follow-up.

However, there are further alternatives to increase detectability of SPIO-labelled cells applied for treatment of tendon disease by high-field as well as low-field MRI, which approach the challenge to distinguish SPIO-induced artefacts from healthy tendon tissue. The latter is due to the similar hypointense appearance of healthy or regenerated tendon tissue and the SPIO-induced artefacts in standard MRI series, which can lead to false-positive or false-negative results in cell detection. In the current study, quantification of signal intensity within putatively SPIO-induced hypointense areas and subsequent comparison to the signal obtained from a healthy tendon structure were helpful to identify a false-positive subjective judgement regarding the presence of SPIO-labelled cells. However, a delineation based on such measurements may not be feasible under all circumstances. At least, the threshold value for distinction of SPIO artefacts and regenerating tendon tissue would likely have to be estimated newly in adaptation to the respective imaging conditions. Furthermore, false-negative judgements are unlikely to be recognized by these means, unless a highly detailed quantitative image analysis of the whole tendon structure would be performed. Aiming to overcome this challenge, own ex vivo studies as well as studies in rabbits have shown that SPIO-induced artefacts and tendon tissue can be differentiated with the help of the so-called magic angle effect [26, 39]. This effect is based on the phenomenon that when tendons are orientated at an angle of approximately 54.7° to the main magnetic field, the T2 relaxation time and signal intensity of tendon tissue increase [40]. Based on that, healthy tendon tissue can then be distinguished from SPIO-induced artefacts. Applying this technique is possible but challenging in the equine dedicated low-field system due to the narrow gantry positioning of the horse [41]. Therefore, it was not performed in the untrained client-owned horses used in the current study but should be attempted in future studies.

Besides offering the possibility of long-term cell tracking, MRI is an ideal modality for imaging of soft tissue regeneration. The study demonstrated that simultaneous monitoring of tendon healing is feasible as the sequences suitable for monitoring of the early healing phase were not influenced by the presence of SPIO-labelled cells. While T1w and T2^*∗*^w GRE sequences were used for cell tracking, T2w FSE sequences were most sensitive to early changes within the tendon lesion. Corresponding to the clinical finding of decreased lameness, a decrease of signal intensity in T2w FSE images indicated improvement of the tendon lesions. This finding corresponds to results obtained in studies investigating induced equine tendon disease [42, 43]. However, in contrast to these studies, we observed no significant differences between T2w FSE images and ultrasonographic images at any examination. This could be either due to the more heterogenous appearance of the natural tendon lesions included in the current study or due to the different follow-up periods. Furthermore, it should be acknowledged that the process of tendon healing might be different in induced tendon lesions compared to natural tendon disease, which represents the most important reason for using natural disease models whenever possible. In either case, it should be considered that even if the appearance of a damaged tendon has normalized in T2w FSE image series or ultrasonography, tendon healing is not completed and should be further monitored by the use of T1w and T2^*∗*^ GRE as well as STIR MRI sequences.

While enabling studies in naturally occurring tendon disease, the use of client-owned horses in this study led to few limitations. On the one hand, no negative control in terms of treatment efficacy was included in the study. Therefore, it should be clearly stated that no conclusions can be drawn regarding the contribution of MSC to tendon healing from this particular study. However, our aim was to gain knowledge on the localization of MSC injected for treatment of tendon disease, which could be achieved by using nonlabelled MSC as a negative control for cell tracking. On the other hand, as discussed above, no histological assessment could be performed; thus, no final statement on the presence of viable MSC within the tendons can be made. Due to these reasons, it still appears important to perform additional studies in the equine model of induced tendon disease, which should be interpreted in consideration of the findings obtained in studies on naturally occurring disease.

## 5. Conclusions

Based on the present findings, MRI is a suitable diagnostic tool for noninvasive, longitudinal cell tracking and simultaneous monitoring of soft tissue regeneration when combining the use of different MRI sequences. Furthermore, our results suggest that allogeneic umbilical cord-derived MSC can safely be used for tendon cell therapy.

## Figures and Tables

**Table 1:** Patient data.

Animal	Breed, usage	Sex	Age (years)	Bodyweight (kg)	Lesion localization (forelimb, level of injury [34])	MSC (foal, passage, and labelling)
1	Warmblood, show jumping	Gelding	10	646	Right, 2a-3a	Foal 2, P3, nonlabelled
2	Warmblood, show jumping	Gelding	9	640	Right, 2a-3a	Foal 2, P3, labelled
3	Pony, pleasure	Gelding	18	420	Left, 1a-3a	Foal 1, P1, labelled
4	Warmblood, dressage	Gelding	4	533	Left, 2a-3a	Foal 1, P2, labelled
5	Thoroughbred, pleasure	Mare	20	440	Right, 2a-3a	Foal 2, P3, nonlabelled
6	Warmblood, dressage	Gelding	14	530	Left, 1a-2a	Foal 1, P2, nonlabelled
7	Warmblood, dressage	Gelding	6	590	Right, 2b-3b	Foal 2, P3, labelled
8	Haflinger, pleasure	Gelding	23	499	Left, 2a-3a	Foal 2, P3, nonlabelled
					Right, 2a-3a	Foal 2, P3, labelled
9	Pony, pleasure	Gelding	22	340	Left, 1a-3a	Foal 1, P1, labelled
					Right, 1a-3a	Foal 1, P1, labelled

**Table 2:** MRI sequences.

Sequence	TR in ms	TE in ms	Flip angle	ST in mm	Gap in mm	FOV in mm	Matrix
T1w GRE	52	8	50°	5	1	171 × 171	256 × 256
T2^*∗*^w GRE	68	13	25°	5	1	171 × 171	256 × 256
T2w FSE	1544	88	90°	5	1	171 × 171	256 × 256
STIR	2336	22	90°	5	1	171 × 171	256 × 256

GRE: gradient echo, FSE: fast spin echo, STIR: short tau inversion recovery, TR: repetition time, TE: echo time, IT: inversion time, ST: slice thickness, and FOV: field of view.
